# Multiple Congenital Segmental Dilatations of Colon: A Case Report 

**Published:** 2012-07-01

**Authors:** Bilal Mirza, Nabi Bux

**Affiliations:** Department of Paediatric Surgery, The Children's Hospital and the Institute of Child Health Lahore, Pakistan

**Keywords:** Multiple congenital segmental dilatations of colon, neonate, rectal atresia

## Abstract

Congenital segmental dilatation of colon (CSDC) is a rare malformation in neonates. A single segmental dilatation of colon is mentioned in the available case reports. Not a single case of multiple CSDC is reported hitherto. We report a case of multiple CSDC associated with cleft lip and palate, and rectal atresia.

## INTRODUCTION

 Congenital segmental dilatation of colon is a rare entity being described mostly in children. It is even rarer in neonates. Some of these patients also have association with anorectal malformations [1]. We report the first case of multiple CSDC in a neonate presenting as neonatal intestinal obstruction. The patient also had associated cleft lip and palate, and rectal atresia. To the best of our knowledge, such a malformation in association with cleft lip and palate, and rectal atresia has not been reported till date.

## CASE REPORT

A 3-day-old male neonate, weighing 2.2 kg, presented with failure to pass meconium, abdominal distension, and bilious emesis since birth. The neonate was a product of consanguineous marriage. He was born by spontaneous vaginal delivery at term in a private hospital. The patient had no history of perinatal problems. The maternal history of polyhydramnios was present. The baby was offered first feed within a few hours of the birth, which was not tolerated. On worsening of the clinical condition over next three days, the baby was referred to our hospital as a case of neonatal intestinal obstruction. In our nursery emergency, the patient was examined and found to have the cleft lip and palate. General physical examination was essentially unremarkable. Abdominal examination revealed visible bowel loops and abdominal distension. On rectal examination, we could not negotiate thermometer beyond 1.5 cm of rectum from the anal verge. Abdominal radiograph showed a big gas shadow in the right abdomen (Fig. 1). A large gas filled bowel loop was noted in the right abdomen on ultrasonography.

**Figure:**
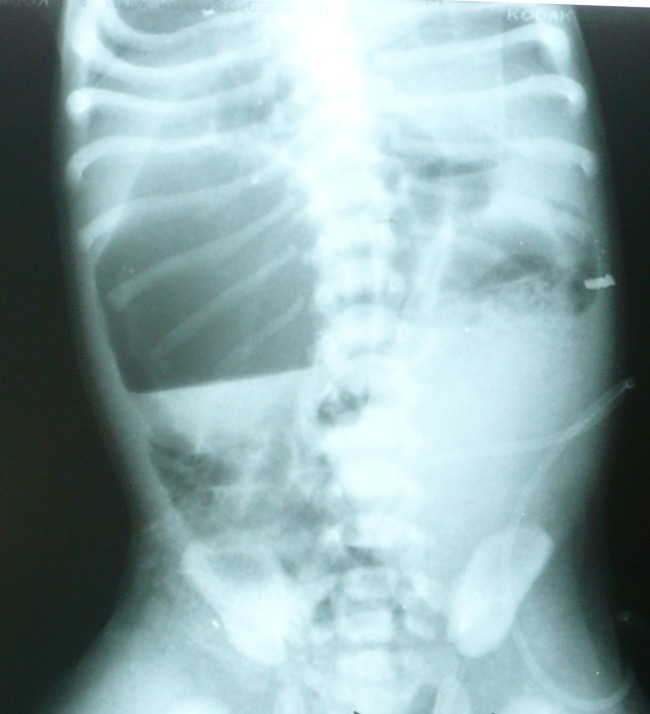
Figure 1: Abdominal radiograph is showing a big gas shadow in the right abdomen and ground glass appearance in the left abdomen. (The gas shadow represents proximal CSDC and ground glass portion may represent distal CSDC- as gas could not have reached the distal CSDC)

A differential diagnosis of rectal atresia resulting in colonic dilatation and/ or associated congenital pouch colon was kept in mind. Exploratory laparotomy was performed after optimization of the patient. There was a CSDC at the level of ascending colon. The colon on either side of the segmental dilatation was of normal caliber and the transition to huge dilatation was abrupt. The segmental dilatation of the colon lacked teniae coli, haustrations, and appendices epiploicae. The mesocolic vessels were dilated and tortuous (Fig. 2). Moreover, there was an out-pouching of the colon present just next to the segmental dilatation (Fig. 3). The transverse colon was normal followed by another similar segmental dilatation of colon at the level of descending colon. The sigmoid colon distal to the second segmental dilatation was of normal caliber and continued behind urinary bladder. Both the CSDC along with that out-pouching were resected and end to end colocolic anastomoses performed. A proximal ileostomy was also fashioned. The proximal CSDC had meconium and gas in it whereas the distal CSDC had more meconium and negligible air contained in it. Before performing distal colocolic anastomosis, a rectal diaphragm was confirmed by passing a nelaton tube through the lumen of sigmoid colon to the rectum. 

**Figure:**
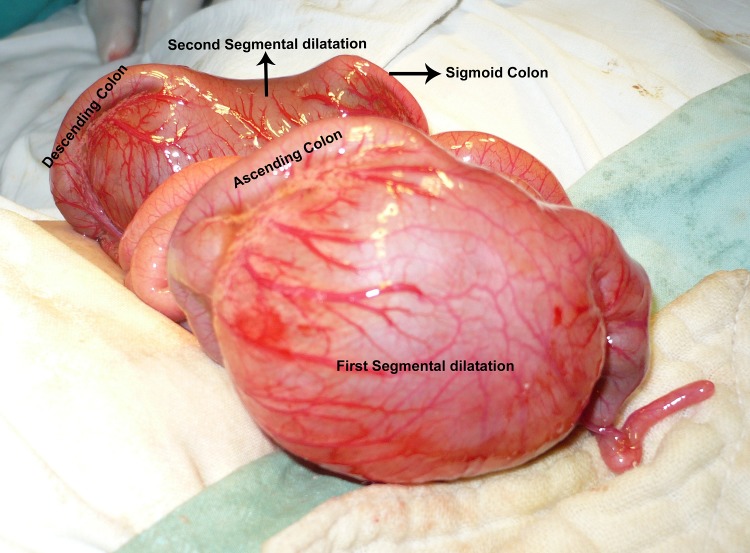
Figure 2: Showing both of the segmental dilatations. The abrupt transition from normal colon to segmental dilatations can be appreciated.

**Figure:**
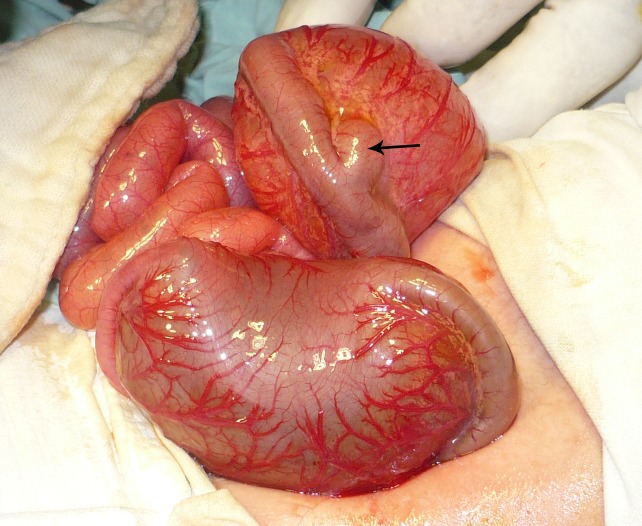
Figure 3: An out-pouching (arrow) from ascending colon just next to the proximal CSDC.

The immediate postoperative recovery was uneventful. On second postoperative day, the ileostomy started moving. The patient was allowed orally on 4th postoperative day. However, a night before the planned discharge the patient aspirated milk. The patient was mechanically ventilated for 4 days during which he developed full blown sepsis. His platelets count dropped to 22000/microliter. Blood culture yielded pseudomonas. The antibiotics were changed according to the culture and sensitivity. Platelets and fresh frozen plasma were transfused. The patient developed petechiae and bruises on all over his body and succumbed to sepsis on 10th postoperative day. The histopathology of the resected CSDC revealed colonic tissue with normal ganglion cells. 

## DISCUSSION

Swenson and Rathauser were first to report their observation on segmental dilatation of colon in a child in 1959 [2]. Since then many case reports on this anomaly were reported in children. There is a paucity of neonatal case reports in literature. Mahadevaiah et al, have reviewed the literature in 2011 and found only 9 cases of CSDC reported in neonates [3]. The presence of segmental dilatation in neonates supports the congenital nature of this poorly understood malformation [3].

CSDC has been reported in association with anorectal malformations, congenital pouch colon, duplex appendix, duplication of cecum, vesical exstrophy, malrotation, duodenal atresia, Meckel’s diverticulum, meningomyelocele, hydrops gallbladder, colonic atresia, and facial defects [1,4-8]. Their association with rectal atresia is however not reported hitherto.

The common symptom in children is constipation; however, in neonates the presentation is usually with neonatal intestinal obstruction [2,8]. Their preoperative diagnosis is not formed in most of the reported cases. These children are often suspected for Hirschsprung’s disease and thus investigated on these lines. Ganglion cells are always present in these patients which differentiate them from those of Hirschsprung’s disease. Rarely, it may present with perforation peritonitis, and volvulus of the sigmoid colon [9,10]. In neonates, CSDC is usually found as an incidental finding; in few cases where it was associated with anorectal malformation, it had simulated congenital pouch colon [1]. We also expected congenital pouch colon as it has been reported in association with rectal atresia [11].

The cardinal features of CSDC include a single large dilatation of colon that lacks teniae coli, haustrations, and appendices epiploicae; and have abundant serosal blood vasculature. The normal colon on both sides of the segmental dilatation abruptly transitioned to the segmental dilatation [2,3]. It usually involves left colon but right colon may be involved. On account of these features, it has got extraordinary resemblance with congenital pouch colon. However, congenital pouch colon is always associated with anorectal malformations, which is not true for CSDC. The presence of normal colon distal to the segmental dilatation and absence of fistulous communication between CSDC and urogenital tract separates them from congenital pouch colon [2,4,8].

The etiology of CSDC is largely based on speculations. Intrauterine vascular accident, failure of or defective organogenesis, strangulation of intestine in the umbilical ring, and defective muscular development are few of proposed theories [3,4]. In the index case, a small out-pouching of colon was also present just next to the first CSDC; this out-pouching might be a developmental error or it could be secondary to the vascular insult during intrauterine life. This out-pouch may have grown later to its present size of CSDC.

Management of this anomaly depends upon the clinical condition of the patient, presentation, surgeon’s experience of dealing such malformations, and association with other malformations. The definitive surgery is resection of the involved segment and end to end anastomosis [8]. In case of critically sick patients, an ileostomy can be fashioned without excision of the segmental dilatation [8]. Other option is resection of the segmental dilatation and colostomy. In case of associated anorectal malformations, stoma formation is always recommended to buy time for anorectoplasty in the second stage. In case of associated congenital pouch colon, both of the lesions can be resected with end ileostomy. [1,4,5]. In the index case, a covering ileostomy was also fashioned on account of multiple colonic anastomoses and rectal atresia.

## Footnotes

**Source of Support:** Nil

**Conflict of Interest:** None

